# Individual and Combined Treatments with Methylated Resveratrol Analogue DMU-214 and Gefitinib Inhibit Tongue Cancer Cells Growth via Apoptosis Induction and EGFR Inhibition

**DOI:** 10.3390/ijms22126180

**Published:** 2021-06-08

**Authors:** Malgorzata Jozkowiak, Marta Dyszkiewicz-Konwinska, Piotr Ramlau, Wieslawa Kranc, Julia Spaczynska, Marcin Wierzchowski, Mariusz Kaczmarek, Jadwiga Jodynis-Liebert, Hanna Piotrowska-Kempisty

**Affiliations:** 1 Department of Toxicology, Poznan University of Medical Sciences, Dojazd 30 St., PL-60-631 Poznan, Poland; malgorzata.jozkowiak@gmail.com (M.J.); pioramlau@gmail.com (P.R.); julaspaczynska@gmail.com (J.S.); liebert@ump.edu.pl (J.J.-L.); 2 Department of Biomaterials and Experimental Dentistry, Poznan University of Medical Sciences, Bukowska 70 St., PL-60-812 Poznan, Poland; m.dyszkiewicz@ump.edu.pl; 3 Department of Anatomy, Poznan University of Medical Sciences, Swiecickiego 6 St., PL-60-781 Poznan, Poland; wkranc@ump.edu.pl; 4 Department of Chemical Technology of Drugs, Poznan University of Medical Sciences, Grunwaldzka 6 St., PL-60-780 Poznan, Poland; mwierzch@ump.edu.pl; 5 Department of Cancer Immunology, Chair of Medical Biotechnology, Poznan University of Medical Sciences, Garbary 15 St., PL-61-866 Poznan, Poland; markacz@ump.edu.pl; 6 Gene Therapy Unit, Department of Cancer Diagnostics and Immunology, Greater Poland Cancer Centre, Garbary 15 St., PL-61-866 Poznan, Poland; 7 Department of Basic and Preclinical Sciences, Institute of Veterinary Medicine, Nicolaus Copernicus University in Toruń, 7 Gagarina St., 87-100 Torun, Poland

**Keywords:** tongue cancer, resveratrol analogue, DMU-214, gefitinib, EGFR inhibitor, apoptosis

## Abstract

The methylated resveratrol analogue 3′-hydroxy-3,4,5,4′-tetramethoxystilbene (DMU-214) has been revealed to exert the anti-cancer activity by a block of the cell cycle at the G2/M phase, apoptosis induction, and metastasis inhibition. These biological events may be involved in crosstalk with the epidermal growth factor receptor (EGFR), which belongs to the ErbB family of receptor tyrosine kinases. Several cancer therapeutic approaches employ small molecules capable of inhibiting tyrosine kinases (e.g., gefitinib). According to more recent reports, combining gefitinib with chemotherapeutics, such as cisplatin, seems to be more effective than monotherapy. The present study aimed to assess the molecular mechanism of the potential anti-proliferative activity of individual and combined treatments with DMU-214 and gefitinib in SCC-25 and CAL-27 human tongue cancer cell lines. We showed for the first time the anti-cancer effects of DMU-214, gefitinib, and their combination in tongue cancer cells triggered via cell cycle arrest, apoptosis induction, and inhibition of the EGFR signaling pathway. The anti-proliferative effects of DMU-214 and gefitinib are also suggested to be related to the EGFR and EGFRP (phosphorylated epidermal growth factor receptor) expression status since we found significantly weaker cytotoxic activity of the compounds tested in SCC-25 cells, which overexpressed EGFR and EGFRP proteins.

## 1. Introduction

Head and neck cancers are important public health issues expressed by the worldwide estimates of the growing number of incidence and mortality rate. According to the International Agency for Research on Cancer, there were over 130,000 new nasopharynx cancer cases and over 370,000 lip and oral cavity tumors in 2020 [1]. The term “head and neck cancers” usually refers to tumors located in the oral cavity, pharynx, larynx, paranasal sinuses, nasal cavity, and salivary glands. Most of them arise in the squamous cell layer of the mucosa lining. Oral cancer represents squamous cell carcinomas growing from premalignant lesions such as leukoplakia and erythroleukoplakia. Among them, tongue cancer is relatively frequent, comprising 42% of all oral malignant tumors. Globally, a rising incidence of tongue cancer has been observed, and the prognosis in diagnosed patients is unsatisfactory. According to the American Cancer Society, there will be 17,960 new cases of tongue tumors, and 2870 patients will die of this disease only in the United States in 2021 [2]. In view of these data, it is a priority to find more accurate diagnostic methods and develop new anti-cancer therapies with higher effectiveness and fewer side effects.

Resveratrol (3,4′,5-trans-trihydroxystilbene) is a natural polyphenol, which occurs in grapes, blueberries, cranberries, peanuts, and various other plants. This phytoalexin, initially isolated from the roots of white hellebore (*Veratrum grandiflorum*), has been reported to exert a wide spectrum of biological activities, including estrogenic, anti-inflammatory, antioxidant, anti-fungal, and anti-tumor ones [3,4]. Despite the fact that the oral absorption of resveratrol is relatively high, its extensive metabolism results in the bioavailability of less than 1% [5]. In this regard, the therapeutic potential of resveratrol is strongly limited due to its poor pharmacokinetics. Based on the available literature, the alternation of the stilbene scaffold of resveratrol is a promising strategy to obtain synthetic derivatives with improved pharmacokinetic properties and stability. The structure-activity relationship studies have revealed that methoxy substitution of hydroxyl groups increases the stability and cytotoxicity of a molecule. Accordingly, the methylated analogue of resveratrol 3′-hydroxy-3,4,5,4′-tetramethoxystilbene (DMU-214) has been revealed to exert a strong antiproliferative effect in breast, liver, and ovarian cancer cells [6,7]. The mechanism of its anti-cancer activity has been elucidated by metastasis inhibition, a block of the cell cycle at the G2/M phase, and apoptosis induction, which are commonly known to be disrupted by the epidermal growth factor receptor (EGFR) signaling pathways [7,8]. 

The poor prognosis of patients with tongue cancer has been shown to be associated with the higher expression of EGFR and its phosphorylated form (EGFRP) in tumor samples [9,10]. EGFR is a well-described transmembrane protein that belongs to the ErbB family of receptor tyrosine kinases. 

Several cancer therapeutic approaches employ small molecules capable of inhibiting tyrosine kinases (e.g., gefitinib, Gef). According to more recent reports, combining Gef with chemotherapeutics, such as cisplatin, seems to be more effective than monotherapy. 

The aim of the present study was to assess the molecular mechanism of the potential anti-proliferative activity of DMU-214, gefitinib, and their combination in SCC-25 and CAL-27 human tongue cancer cell lines. We analyzed their effect on apoptosis induction, cell cycle, and the expression profile of genes and proteins involved in EGFR signaling pathways. 

## 2. Results

### 2.1. Effect of DMU-214, Gef, and the Combination of DMU-214 and Gef on the Viability of CAL-27 and SCC-25 Tongue Cancer Cell Lines

**Figure 1:**
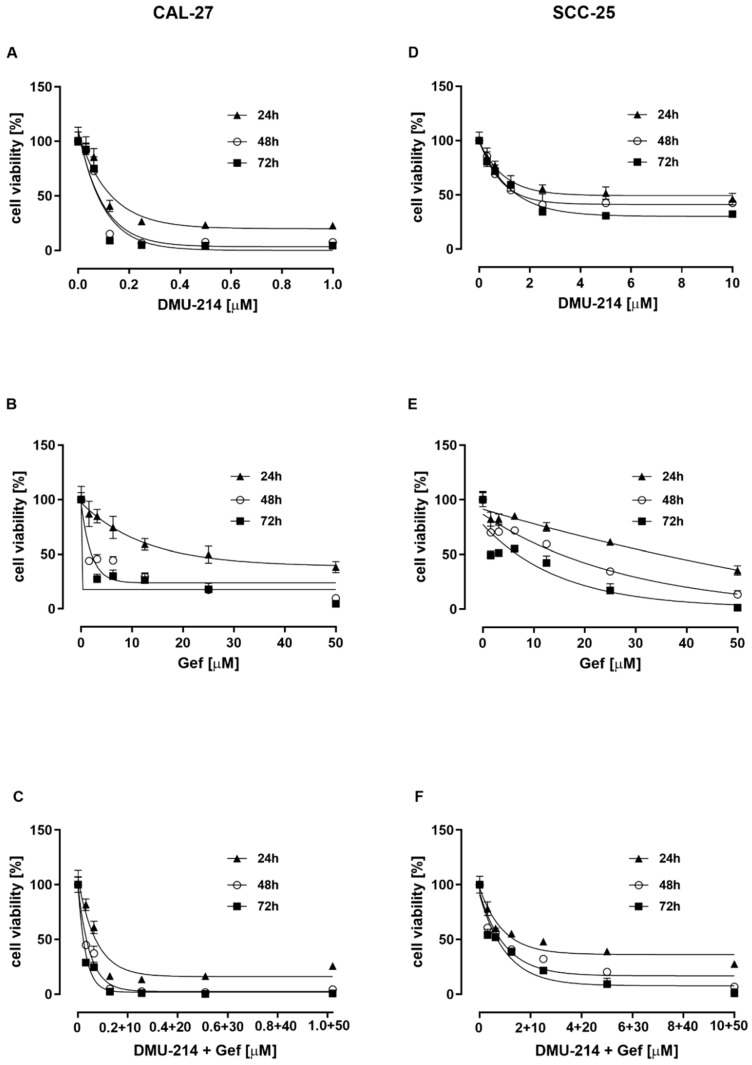
The cytotoxic activity of DMU-214, Gef, and the combination of DMU-214 and Gef in CAL-27 and SCC-25 cell lines. The viability of CAL-27 cells was determined after 24, 48, and 72 h incubation with DMU-214 (0–1 μM) (**A**), Gef (0–50 μM) (**B**), and the mixture of DMU-214 (0–1 μM) and Gef (0–50 μM) (**C**). The viability of the SCC-25 cell line was assessed following 24, 48, and 72 h incubation with DMU-214 (0–10 μM) (**D**), Gef (0–50 μM) (**E**), and the mixture of DMU-214 (0–10 μM) and Gef (0–50 μM) (**F**). Results of three independent replicates are presented as mean ± SD.

To investigate the cytotoxic activity of DMU-214, Gef, and the combination of DMU-214 and Gef in SCC-25 and CAL-27 tongue cancer cells, the MTT assay was performed. A significant decrease in viability of both cell lines exposed to all treatments for 24 h, 48 h, and 72 h was shown in Figure 1 and Table 1. CAL-27 cells were more susceptible to DMU-214, Gef, and their mixture as compared to the SCC-25 cell line. DMU-214 was reported to exert higher cytotoxic effects than Gef in both cell lines tested. Simultaneously, combining DMU-214 and Gef was shown to increase their cytotoxicity as compared to the individual treatments (Figure 1).

### 2.2. Effect of DMU-214, Gef, and the Combination of DMU-214 and Gef on Apoptosis and Necrosis Induction

**Figure 2:**
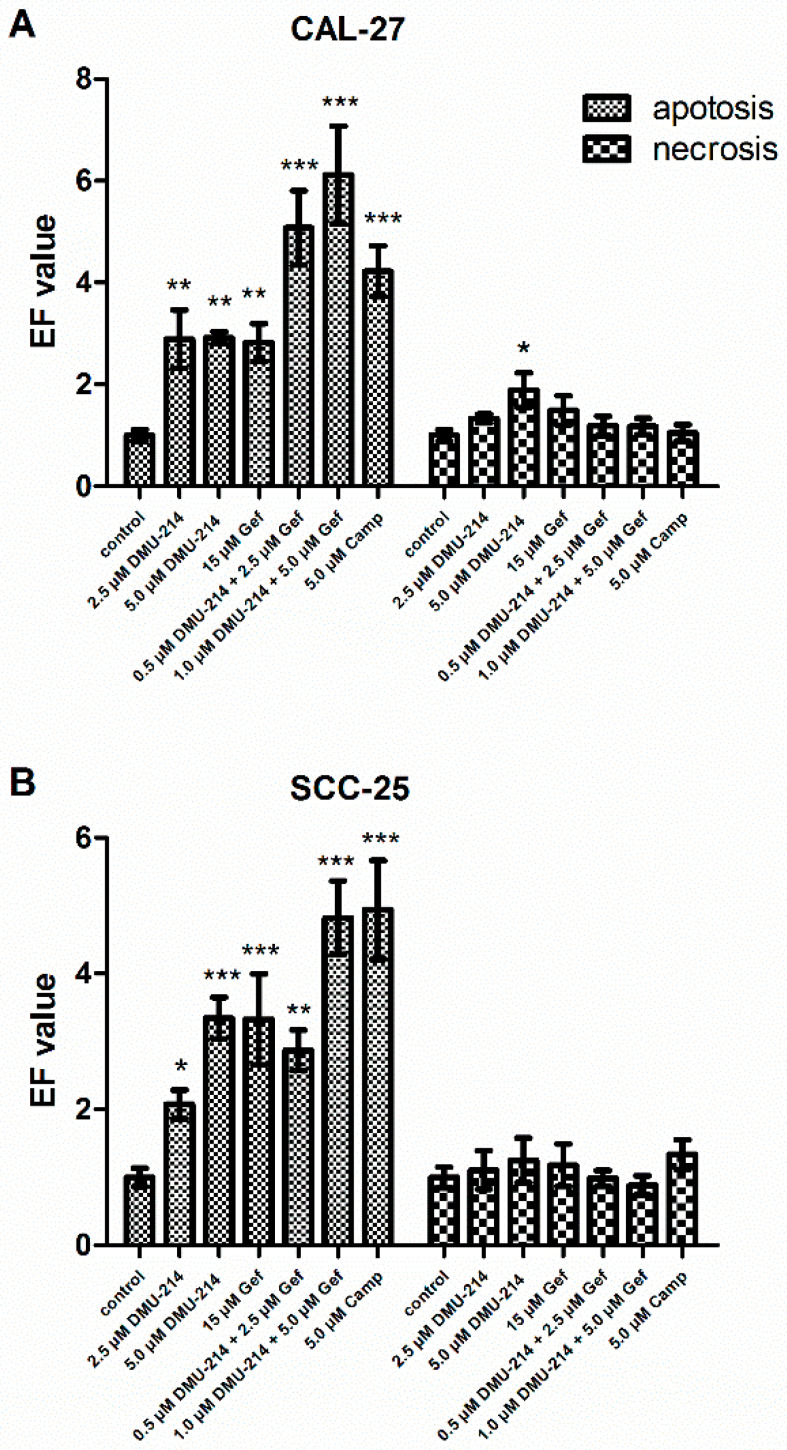
Effect of DMU-214, Gef, and the combination of DMU-214 and Gef on apoptosis and necrosis induction in CAL-27 and SCC-25 cell lines. CAL-27 and SCC-25 cells were treated for 24 h with vehicle, DMU-214 (2.5 µM and 5.0 µM), Gef (15 µM), and the mixtures of both compounds: (i) 0.5 µM DMU-214 and 2.5 µM Gef, (ii) 1.0 µM DMU-214 and 5.0 µM Gef. Apoptosis and necrosis assay was performed by ELISA test and expressed as an enrichment factor (EF) in CAL-27 (**A**) and SCC-25 (**B**) cell lines. Camptothecin (Camp) was used as a positive control for apoptosis. Results of three independent replicates are presented as mean ± SD. *** *p* < 0.001, ** *p* < 0.01, * *p* < 0.05 indicate a significant difference from the control.

The induction of apoptosis and necrosis was assayed by the Cell Death Detection ELISA^PLUS^ test. The pro-apoptotic and -necrotic activities of the compounds tested were expressed as the enrichment factors (EFs). Camptothecin was used as a positive control for apoptosis induction and caused an increase in the nucleosome level in CAL-27 and SCC-25 lysates, EF = 4.22 ± 0.49 and EF = 4.94 ± 0.72, respectively (Figure 2A,B). The highest pro-apoptotic effect was reported after exposure of CAL-27 cell line to the mixture of compounds tested in the concentrations of 1.0 µM DMU-214 and 5.0 µM Gef as well as 0.5 µM DMU-214 and 2.5 µM Gef, EF = 6.11 ± 0.96 and EF = 5.07 ± 0.73, respectively (Figure 2A). The increased EF values were also shown after 2.5 μM and 5.0 μM DMU-214 treatments. However, as compared to both combinations of DMU-214 and Gef, the pro-apoptotic activity of DMU-214 was less pronounced not only in the concentration of 2.5 μM (EF = 2.89 ± 0.57), but also 5 μM (EF = 2.91 ± 0.11). Correspondingly, the exposure to Gef in the concentration of 15 μM also caused an increase in the level of the nucleosomes in CAL-27 lysates (EF = 2.82 ± 0.37), but to a lesser extent than the mixture of DMU-214 and Gef.

The statistically significant pro-apoptotic effects of all treatments were also shown in the SCC-25 tongue cancer cell line (Figure 2B). Nevertheless, this up-regulation was pronounced the most after treatment with the mixture of both compounds in the concentration of 1.0 µM DMU-214 and 5.0 µM Gef (EF = 4.82 ± 0.54), as compared to control. Exposure of SCC-25 cells to the combination of 0.5 µM DMU-214 and 2.5 µM Gef, DMU-214 (2.5 μM and 5 μM), and Gef (15 μM), resulted in a lower pro-apoptotic activity; EF = 2.87 ± 0.29, EF = 2.077 ± 0.21 and EF = 3.34 ± 0.30, EF = 3.32 ± 0.66, respectively.

The number of necrotic cells was also evaluated; a statistically significant difference was noted only in the CAL-27 cell line after treatment with DMU-214 in the highest concentration tested (Figure 2A).

### 2.3. Effect of DMU-214, Gef, and the Combination of DMU-214 and Gef on Caspase-8, -9, -3/7 Activation in CAL-27 and SCC-25 Cell Lines

**Figure 3:**
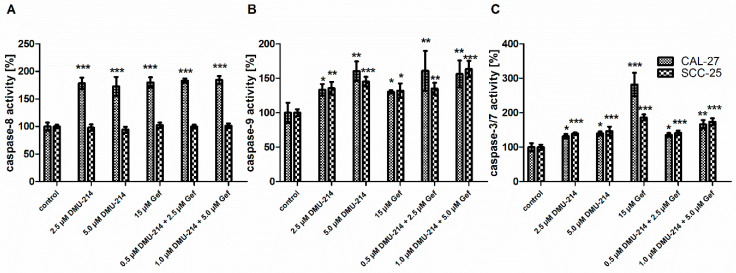
Effect of DMU-214, Gef, and the combination of DMU-214 and Gef on caspases activity in CAL-27 and SCC-25 cell lines compared to the vehicle-treated control. The casp-8 (**A**), -9 (**B**) and -3/7 (**C**) activities were determined by Caspase-Glo^®^-8, -9, -3/7 assay. Results of three independent replicates are presented as mean ± SD. *** *p* < 0.001, ** *p* < 0.01, * *p* < 0.05 indicate a significant difference from the control.

As shown in Figure 3A, the increase in the activation of caspase-8 by ~80% occurred in the CAL-27 cell line following all treatments. Conversely, in the SCC-25 cell line, no effects of DMU-214, Gef, and the combination of DMU-214 and Gef on caspase-8 activation were observed. 

The induction of caspase-9 activity was found in both tongue cancer cell lines after exposure to all compounds tested (Figure 3B). DMU-214 (2.5 μM) and Gef (15 μM) applied alone were shown to up-regulate the activation of caspase-9 in CAL-27 cells by ~35% as compared to control. Concomitantly, the higher concentration of DMU-214 (5.0 μM) and both combinations of Gef and DMU-214 caused an increase in the activity of caspase-9 by ~55%. In the SCC-25 cell line, the up-regulated activity of this initiator caspase by ~30–35% occurred after treatments with DMU-214 (2.5 μM and 5.0 μM), Gef (15 μM), and the combination of DMU-214 (0.5 μM) and Gef (2.5 μM) as compared to control. The higher concentration of the latter one induced the caspase-9 activity by ~50% (Figure 3B). 

After exposure of both cell lines to DMU-214 and combinations of DMU-214 and Gef in all concentrations tested, the increase in the activation of caspase-3/7 by ~40–45% occurred in comparison to control (Figure 3C). Additionally, treatment with Gef (15 μM) resulted in the up-regulation of the activity of caspase-3/7 by ~180% and ~80% in CAL-27 and SCC-25 cells, respectively.

### 2.4. Cell Cycle Analysis

**Figure 4:**
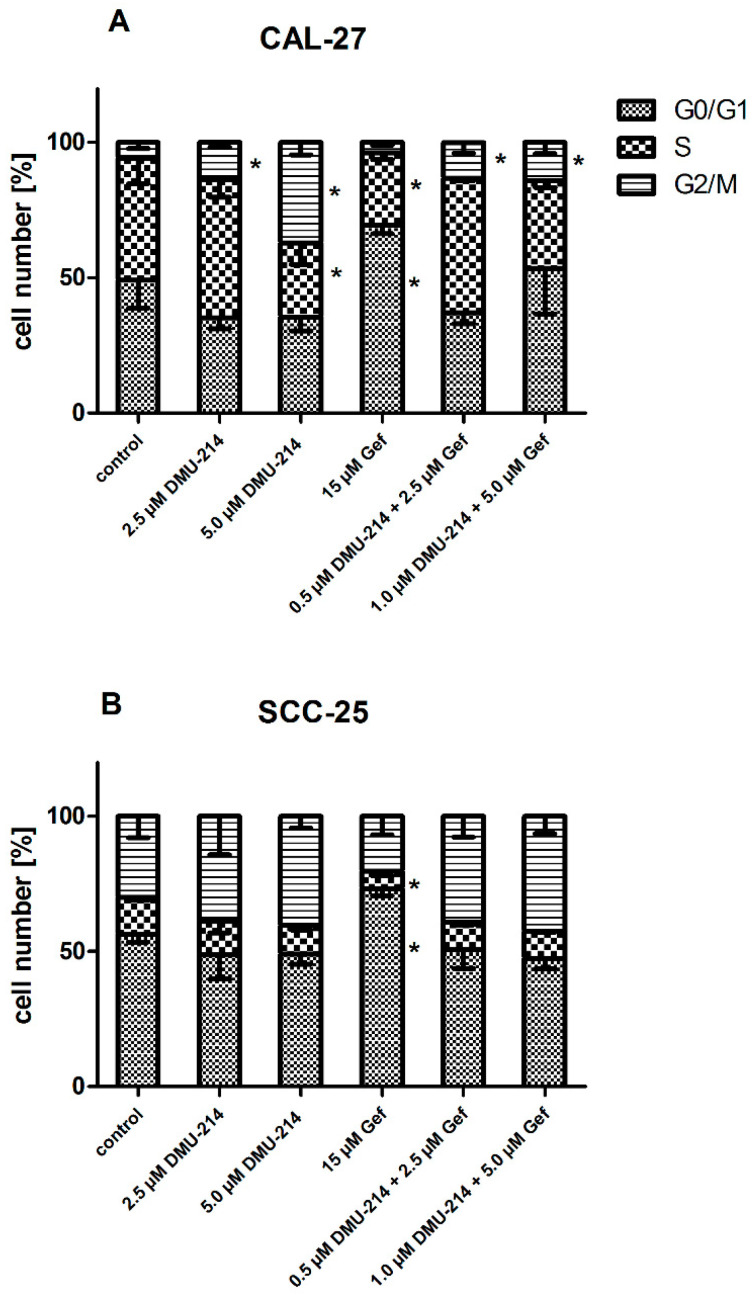
Effect of DMU-214, Gef, and the combination of DMU-214 and Gef on cell proliferation compared to the vehicle-treated control. Cell cycle phase distribution was analyzed by flow cytometry in CAL-27 (**A**) and SCC-25 (**B**) cell lines treated for 24 h with the vehicle, DMU-214 (2.5 µM and 5.0 µM), Gef (15 µM), and the mixtures of both compounds: (i) 0.5 µM DMU-214 and 2.5 µM Gef, (ii) 1.0 µM DMU-214 and 5.0 µM Gef. Results of three independent replicates are presented as mean ± SD. * *p* < 0.05 indicates a significant difference from the control.

Flow cytometry analysis demonstrated that CAL-27 cells were arrested in the G2/M phase with both concentrations of DMU-214 applied alone and in combinations with Gef (Figure 4A). Treatment with DMU-214 (5 μM) also resulted in a decreased CAL-27 cell number in S phase as compared to the untreated controls. The exposure of CAL-27 and SCC-25 cell lines to Gef (15 μM) caused a cell cycle arrest at G0/G1 phase, while the number of cells in the S phase was decreased (Figure 4A,B).

### 2.5. Protein Expression Analyses 

**Figure 5:**
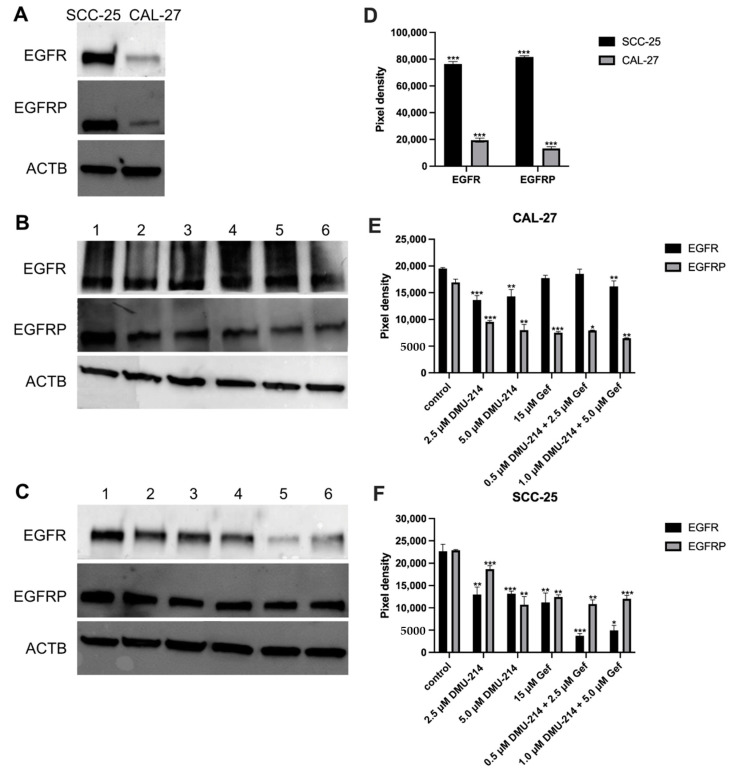
Western blot analysis of EGFR and EGFRP native expression pattern (**A**) as well as the effect of DMU-214, Gef, and the combination of DMU-214 and Gef on the EGFR and EGFRP expression profile in CAL-27 (**B**) and SCC-25 (**C**) cell lines. Cells were treated with: lane 1—control, lane 2—2.5 μM DMU-214, lane 3—5 μM DMU-214, lane 4—15 μM Gef, lane 5—0.5 μM DMU-214 + 2.5 μM Gef, lane 6—1.0 μM DMU-214 + 5.0 μM Gef. ACTB (β-Actin) was used as a loading control. Densitometric studies were used to analyze the level of EGFR and EGFRP in native expression pattern (**D**), as well as in CAL-27 (**E**) and SCC-25 (**F**) cells treated with a vehicle or DMU-214, Gef, and the combination of DMU-214 and Gef. Results of three independent replicates are presented as mean ± SD. *** *p* < 0.001, ** *p* < 0.01, * *p* < 0.05 indicate a significant difference from the control.

Western blot was employed to analyze the effect of compounds tested on the level of EGFR and EGFRP proteins in SCC-25 and CAL-27 cells. Initially, the investigation of the native expression profile of EGFR and EGFRP proteins in both tongue cancer cell lines was performed. As can be seen from Figure 5A, the level of EGFR and EGFRP proteins was significantly lower in CAL-27 cells as compared to the SCC-25 ones. 

Exposure of CAL-27 cells to DMU-214 in both concentrations tested and the combination of DMU-214 (1 μM) and Gef (5 μM) resulted in a slightly decreased EGFR protein expression as compared to control (Fig. 5B). There were no significant changes in EGFR protein level after treatments with Gef (15 μM) as well as the combination of DMU-214 (0.5 μM) and Gef (2.5 μM). 

In CAL-27 cells exposed to the individual and combined treatments, a down-regulation in the expression of EGFRP protein by about 50% was shown as compared to control (Figure 5B). 

Exposure of SCC-25 cell line to DMU-214 and Gef in all concentrations tested resulted in down-regulation by ~50% of EGFR protein expression as compared to control (Figure 5C). The treatment with both combinations of DMU-214 and Gef caused a decreased EGFR protein level by ~80%.

A reduction in the expression of EGFRP protein by ~50% in the SCC-25 cell line exposed to all compounds tested was shown, except for the lower concentration of DMU-214 (↓ ~20%). 

### 2.6. Effect of DMU-214, Gef, and the Combination of DMU-214 and Gef on the Expression of EGFR Signaling Pathway-Related Genes

**Figure 6:**
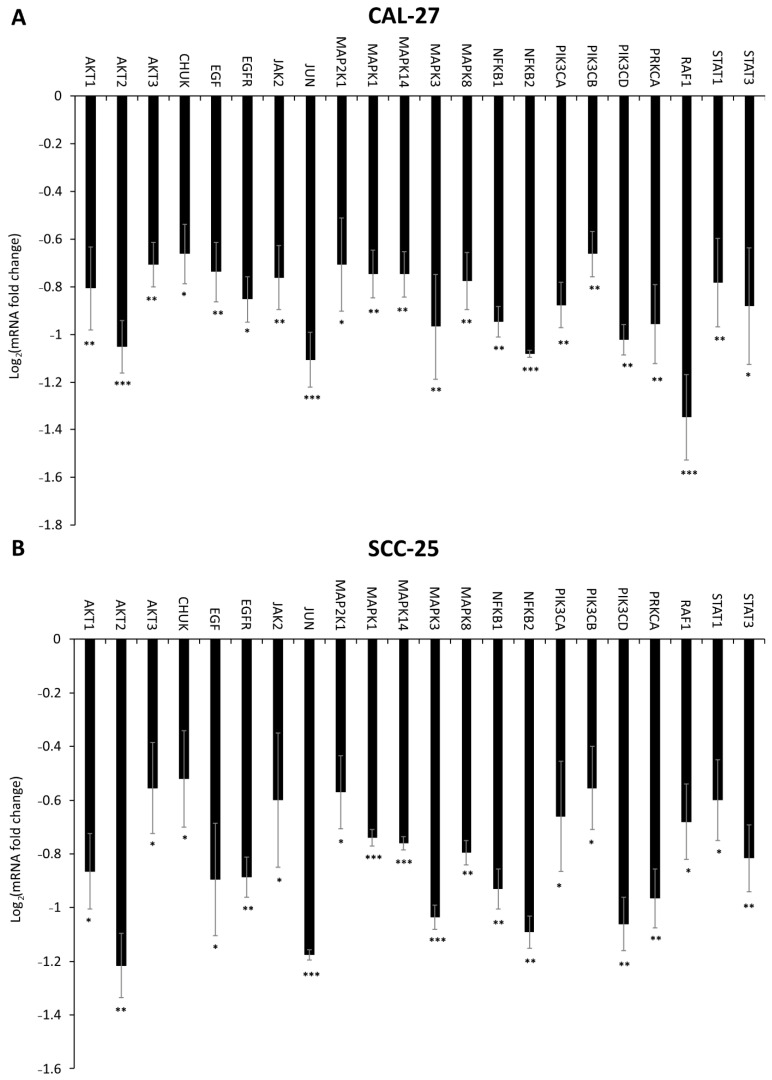
The effect of DMU-214 (5 μM) on the expression of EGFR signaling pathway-related genes in CAL-27 (**A**) and SCC-25 (**B**) cell lines. Results of three independent replicates are presented as mean ± SD. *** *p* < 0.001, ** *p* < 0.01, * *p* < 0.05 indicate a significant difference from the control.

**Figure 7:**
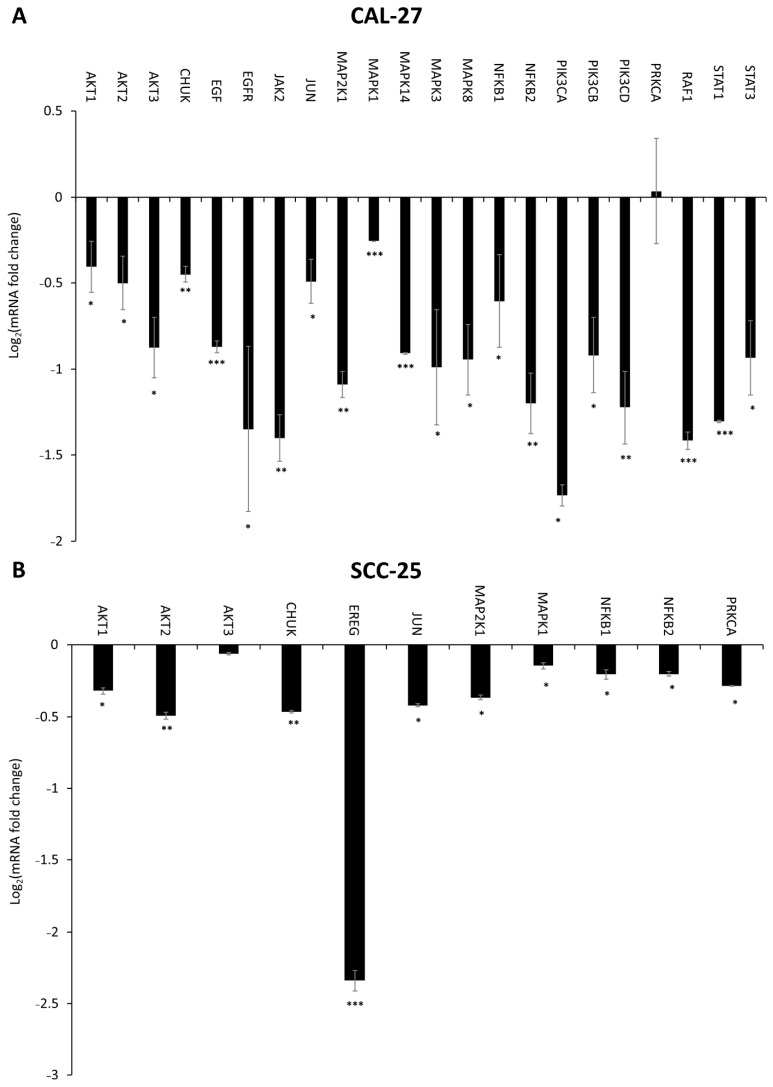
The effect of Gef (15 μM) on the expression of EGFR signaling pathway-related genes in CAL-27 (**A**) and SCC-25 (**B**) cell lines. Results of three independent replicates are presented as mean ± SD. *** *p* < 0.001, ** *p* < 0.01, * *p* < 0.05 indicate a significant difference from the control.

**Figure 8:**
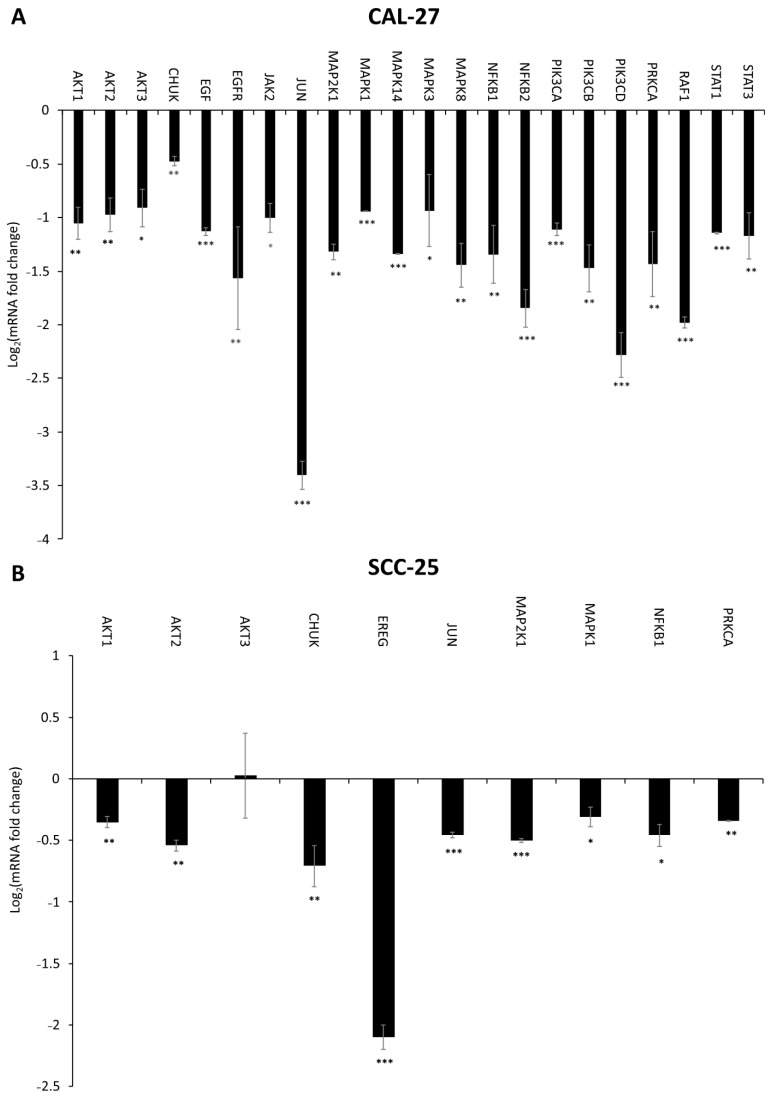
The effect of the combination of DMU-214 (1.0 μM) and Gef (5.0 μM) on the expression of EGFR signaling pathway-related genes in CAL-27 (**A**) and SCC-25 (**B**) cell lines. Results of three independent replicates are presented as mean ± SD. *** *p* < 0.001, ** *p* < 0.01, * *p* < 0.05 indicate a significant difference from the control.

The RT-qPCR was conducted to analyze the expression profile of EGFR signaling pathway-related genes in CAL-27 and SCC-25 cell lines (Figure 6, Figure 7 and Figure 8). Significant changes in the expression pattern of 22 genes were found in CAL-27 and SCC-25 cell lines treated with 5 μM DMU-214 (Figure 6A,B). A down-regulation in the level of the following transcripts: *AKT1*, *AKT2*, *AKT3*, *CHUK*, *EGF*, *EGFR*, *JAK2*, *JUN*, *MAP2K1*, *MAPK1*, *MAPK14*, *MAPK3*, *MAPK8*, *NFĸB1*, *NFĸB2*, *PIK3CA*, *PIK3CB*, *PIK3CD*, *PRKCA*, *RAF1*, *STAT1*, and *STAT3* was shown as compared to control. 

The exposure of CAL-27 cell line to Gef (15 μM) resulted in a marked decrease in mRNA expression of *AKT1*, *AKT2*, *AKT3*, *CHUK*, *EGF*, *EGFR*, *JAK2*, *JUN*, *MAP2K1*, *MAPK1*, *MAPK14*, *MAPK3*, *MAPK8*, *NFKB1*, *NFKB2*, *PIK3CA*, *PIK3CB*, *PIK3CD*, *PRKCA*, *RAF1*, *STAT1*, and *STAT3* (Figure 7A). In SCC-25 cells treated with Gef (15 μM), a down-regulation in transcripts level of *AKT1*, *AKT2*, *AKT3*, *CHUK*, *EREG*, *JUN*, *MAP2K1*, *MAPK1*, *NFKB1*, *NFKB2*, and *PRKCA* was observed in comparison to control (Figure 7B).

In CAL-27 cells exposed to the combination of DMU-214 (1.0 μM) and Gef (5.0 μM), we observed a lower expression of *AKT1*, *AKT2*, *AKT3*, *CHUK*, *EGF*, *EGFR*, *JAK2*, *JUN*, *MAP2K1*, *MAPK1*, *MAPK14*, *MAPK3*, *MAPK8*, *NFKB1*, *NFKB2*, *PIK3CA*, *PIK3CB*, *PIK3CD*, *PRKCA*, *RAF1*, *STAT1*, and *STAT3* as compared to control (Figure 8A). Concomitantly, in the SCC-25 cell line exposed to the mixture of DMU-214 and Gef, the decreased level of *AKT1*, *AKT2*, *AKT3*, *CHUK*, *EREG*, *JUN*, *MAP2K1*, *MAPK1*, *NFKB1*, and *PRKCA* transcripts was noted (Figure 8B).

## 3. Discussion

In the present study, the inhibitory effects of DMU-214, Gef, and their combination against CAL-27 and SCC-25 human tongue cancer cells were evaluated. We showed that both monotherapy and combined treatments caused a significant decrease in viability of studied cell lines. However, the cytotoxic effects of tested compounds were much more pronounced in CAL-27 cells as compared to the SCC-25 ones. The overexpression of *EGFR* is commonly observed in tongue cancer and is related to the increased resistance to treatment with chemotherapeutics [11,12]. We observed a significantly higher level of EGFR and EGFRP proteins in less sensitive SCC-25 cell line as compared to CAL-27 one. Hence, the cytotoxic effects of DMU-214, Gef, and the combination of both are suggested to be associated with the EGFR and EGFRP expression status.

According to the recent reports, the cytotoxic activity of DMU-214 and Gef applied separately induces apoptosis in several human cancer cell lines [6,7,13]. In this study, the compounds tested applied individually and in combination were also reported to exert significant pro-apoptotic effects in both cell lines, as evidenced by the high EF values. Hence, we suggest that the cytotoxicities of DMU-214 and Gef are associated with their ability to induce apoptosis in tongue cancer cells. Helal et al. (2015) have reported that the combination of Gef with common chemotherapeutic drug such as cisplatin was more effective in cancer treatment than monotherapy [14]. It is in agreement with our results since we documented a distinctly stronger pro-apoptotic activity of the combined treatment with Gef and DMU-214 than with single agent treatments in both tested cell lines. 

It is well known that caspase-9 plays a crucial role in the activation of intracellular pathway of apoptosis. In the present study, the augmented activation of this mitochondrial caspase was observed in both CAL-27 and SCC-25 cell lines treated with DMU-214 and Gef separately and in combination. Therefore, we suggest their ability to induce intrinsic mode of apoptosis. Caspase-8 is a well-known initiator of the caspase cascade in the extrinsic death pathway. Sekiguchi et al. (2019) have revealed that Gef caused an increase in the activity of caspase-8 in human fibrosarcoma HT1080 and lung carcinoma A549 cell lines as well as mouse embryonic fibroblasts (MEFs) [15]. Concomitantly, our previously published data have shown that treatment of human ovarian cancer cell lines A-2780 and SKOV-3 with DMU-214 resulted in the up-regulated activity of caspase-8, which was accompanied by the induction of the receptor-mediated apoptosis pathway [7]. In this study, we also observed the increased activity of caspase-8 in more sensitive CAL-27 cells after treatment with both compounds alone as well as their combination. Since the augmentation in the activity of caspases-8 and -9 was shown in the CAL-27 cell line, we suggest that both receptor and mitochondrial pathways contribute to the apoptotic response to DMU-214 and Gef. Furthermore, the greater vulnerability of the CAL-27 cell line might be associated with the triggering of both modes of apoptosis by tested compounds. Initiator caspases are commonly known to cleave and activate downstream executive caspases-3 and -7, leading eventually to cell death. In CAL-27 and SCC-25 cell lines treated with DMU-214 and Gef, we also showed the increased activity of casp-3/7 that marks the effector phase of apoptosis. 

One of the mechanisms of the anti-cancer activities of DMU-214 or Gef seems to be related to its effect on the cell cycle [7,16]. We showed that Gef caused a block of the cell cycle progression in the G0/G1 phase in CAL-27 and SCC-25 cell lines. These observations are in agreement with the previous findings that Gef inhibits the proliferation of pancreatic cancer cells as a consequence of cell cycle arrests in G0/G1 and G2/M [16]. Concomitantly, cell accumulation in the G2/M phase was shown to be related to the anti-cancer activities of DMU-214 and its parent compound DMU-212 in ovarian cancer cells [7,17]. In the present study, DMU-214 and the combination of DMU-214 and Gef caused an increase in the number of CAL-27 cells in the G2/M phase, while no changes in SCC-25 were shown. Therefore, we suggest that the greater sensitivity of the CAL-27 cell line to the compounds tested might also be associated with their ability to inhibit the cell cycle in the G2/M phase in these cells. 

EGFR is commonly known to be implicated in cell proliferation, survival, migration, and differentiation and, hence, its activation plays an important role in cancer biology. EGFR can be activated through EGF binding, followed by receptor dimerization, trans-autophosphorylation, and the recruitment of signaling proteins or adaptors [18].

We found that DMU-214 and its combination with Gef caused a significant decrease in the mRNA level of *EGF* and *EGFR* accompanied by the reduced expression of EGFR protein as well as its phosphorylated form (EGFRP) in more sensitive CAL-27 cells. Although we did not find any changes in transcripts level of *EGF* and *EGFR* in the SCC-25 cell line, the decreased expression of EGFR and EGFRP proteins was noted. Moreover, treatment of SCC-25 cells with Gef and its combination with DMU-214 resulted in a down-regulated mRNA level of *EREG*, which as a ligand of EGFR is known to contribute to the activation of the receptor and, therefore, cancer cells proliferation [19]. Consequently, we suggest that DMU-214 and its combination with Gef evoke anti-proliferative effects in both CAL-27 and SCC-25 cell lines via inhibition of the expression of EGFR and its phosphorylated form EGFRP. Ligand binding to EGFR and the subsequent autophosphorylation of the receptor is known to activate signaling cascades mediated by Ras/Raf/MAP kinase, JAK2/STAT3 phosphoinositide-3-kinase (PI3K)/AKT [18,20]. Hence, we assessed the expression profile of genes involved in these signaling pathways to clarify the contribution of EGFR inhibition into the mechanism of the anti-cancer activity of the compounds tested in CAL-27 and SCC-25 cells. In the present study, we noted a decreased expression of the *RAF-1* gene in both cell lines treated with DMU-214 and in more sensitive CAL-27 cells after exposure to Gef as well as its combination with DMU-214. Helal et al. (2015) have revealed that the anticancer activity of Gef in combination with one of the chemotherapy regimens was related to a decrease in the expression of *RAF-1* and inhibition of RAF/EGFR-activated signaling pathway in tongue cancer cells [13]. Our results are in agreement with these findings since we showed reduced expression of *RAF-1*, EGFR and EGFRP accompanied by the suppressed proliferation of CAL-27 and SCC-25 cell lines treated with compounds tested. RAF is known to activate mitogen-activated protein kinase kinase (MAPKK) and subsequently phosphorylation of MAPK, which are the frequent events in cancerogenesis [21,22]. In this study, the downregulation of genes related to the MAPKK signaling pathway after exposure to all compounds tested was presented. In the SCC-25 cell line, we found a significant decrease in *MAP2K1*, *MAPK1*, and *JUN* transcript levels after Gef treatment and its combination with DMU-214. Furthermore, the exposure of SCC-25 cells to DMU-214 caused lower expression of *MAPK14*, *MAPK3*, and *MAPK8* genes. Concomitantly, in more sensitive CAL-27 cells, we revealed a significant decrease in the expression of *MAP2K1*, *MAPK1*, *MAPK14*, *MAPK3*, *MAPK8*, and *JUN* after exposure to Gef and the combination of DMU-214 and Gef. For this reason, we suggest that inhibited expression of genes involved in the MAPK signaling pathway triggered by the tested compounds might be involved in the mechanism of their anti-proliferative activities in tongue cancer cells. Recently, crosstalk between MAPK and JAK2/STAT3 pathways in melanoma cells has been revealed [23]. Janus-activated kinase 2 (JAK2) is a protein tyrosine kinase involved in various cytokine receptor signaling pathways. Once JAK2 phosphorylates the STATs (Signal Transducers and Activators of Transcription), they got activated and regulate the transcription of genes involved in cell proliferation and survival [20,24]. In SCC-25 cells, a decreased expression of *JAK2* was noted after exposure to DMU-214. The separate treatments of these cells with DMU-214 and Gef caused a down-regulation in the level of *STAT1* and *STAT3* transcripts. In more sensitive CAL-27 cell line, we observed a decreased expression of *JAK2*, *STAT1*, and *STAT3* after exposure to DMU-214, Gef, and the combination of both. Zhao et al. (2020) have shown that the simultaneous knockout in JAK2/STAT3 and MAPK signaling cascades was an effective anticancer approach for melanoma treatment [23]. It is in agreement with our results since the inhibition of the expression of genes involved in both JAK2/STAT3 and MAPK pathways triggered by the tested compounds was accompanied by the decreased viability of CAL-27 and SCC-25 cells. Therefore, we suggest that inhibited expression of genes involved in JAK2/STAT3 and MAPK signaling pathways might be related to the anticancer activities of DMU-214 and Gef in tongue cancer cells. 

The EGFR/PI3K/AKT cascade is an intracellular signaling pathway of great importance in cell proliferation and survival [25]. PI3Ks are a family of lipid kinases divided into three classes, of which class I is known to play a key role in cancer development. Class I PI3Ks are consisted of two subunits, the regulatory p85, and the catalytic p110 ones. The latter is encoded by *PIK3CA*, *PIK3CB*, and *PIK3CD* genes [25,26]. In the present study, we found a decreased mRNA level of *PIK3CA*, *PIK3CB*, and *PIK3CD* in more sensitive CAL-27 cell line treated with separate and combined compounds tested, and in SCC-25 cells, however, only after exposure to DMU-214. Protein kinase 3 alpha (PRKCA) has been revealed to activate PI3K [27]. Simultaneously, PI3K is commonly known to phosphorylate and further activate AKT1 and AKT2 kinases [25,28]. Our results showed a suppressed expression of *PRKCA*, *AKT1*, and *AKT2* genes in CAL-27 and SCC-25 cell lines after single agents and combined treatments. The PI3K/AKT signaling pathway seems to be one of the most commonly activated drivers of head and neck cancers [25]. Since we showed the ability of DMU-214 and Gef to reduce the expression of genes related to the PI3K/AKT pathway, the tested compounds might be suggested as candidates for tongue cancer therapeutics.

It is well known that AKT contributes to the activation of the anti-apoptotic NFκB pathway [25,29]. In our study, we found significantly decreased expression of *NFκB1* and *NFκB2* genes in both cell lines treated separately with DMU-214 and Gef. The combination of DMU-214 and Gef also caused a down-regulation in mRNA level of *NFκB1* and *NFκB2* in more sensitive CAL-27 cell line, while in SCC-25, a decreased expression of *NFκB1* was noted. NFκB pathway is induced via phosphorylation and activation of IKKα, which with IKKβ then phosphorylates the NF-κB inhibitor IκBα [25,29]. In both cell lines treated with DMU-214, Gef, and the combination of both, we showed a suppressed expression of the conserved helix-loop-helix ubiquitous kinase (CHUK), which is a part of the IκB complex and plays a pivotal role in the regulation of NFκB transcription [30]. Since NFκB is known to be involved in cancer progression, we suggest that the anticancer activities of the tested compounds might also be associated with the inhibition of the NFκB signaling pathway. 

A few studies have shown the anti-proliferative activity of DMU-214 in ovarian, breast, and liver cancer cells [6,7]. Gef, a small molecule EGFR tyrosine kinase inhibitor, has been reported to be commonly used as an experimental treatment in tongue cancer [31]. In the present study, we showed for the first time the cytotoxic effects of DMU-214, Gef, and their combination in tongue cancer cell lines via cell cycle arrest, apoptosis induction, and inhibition of the EGFR signaling pathway. It is well known that suppression of EGFR is still one of the most desired anti-cancer strategies. Therefore, we suggest that both tested compounds might be considered as potential therapeutics for tongue cancer treatment. Furthermore, our results indicate that the anti-proliferative effects of DMU-214 and Gef may be related to the EGFR and EGFRP expression status since we have observed distinctly weaker cytotoxic activity of the compounds tested in SCC-25 cells, which overexpressed EGFR and EGFRP proteins. However, to verify our findings, further studies (e.g., in an animal model) are needed to be conducted.

## 4. Materials and Methods

### 4.1. Chemicals and Reagents

DMU-214 was synthesized as described elsewhere [6]. The identity and purity of the compound were confirmed using NMR and LC–MS. Gefitinib was obtained from Sigma-Aldrich Co. (Darmstadt, Germany). The Cell Death Detection ELISA^PLUS^ kit was provided by Roche Diagnostics (Mannheim, Germany). Caspase-Glo^®^ -8, -9, -3/7 assay kits were purchased from Promega Co. (Madison, WI, USA). Super Signal West Pico PLUS Chemiluminescent Substrate was purchased from Thermo Scientific (Waltham, MA, USA). Trans-Blot Turbo Mini 0.2 µm PVDF Transfer Packs set, Quick Start Bradford Protein A, SsoAdvanced Universal Probes Supermix were provided by Bio-Rad (Hercules, CA, USA). All other materials were from Sigma-Aldrich Co. (St. Louis, MO, USA) unless otherwise stated.

### 4.2. Cell Culture and Cell Viability

SCC-25 and CAL-27 tongue cancer cell lines were purchased from the European Type Culture Collection (Sigma-Aldrich Co., St. Louis, MO, USA). The cells were cultivated in the cell culture flasks in an incubator under optimal conditions (temperature: 37 °C, humidity: 95%, carbon dioxide content: 5%). The SCC-25 cell line was cultured in 1:1 mixture of phenol red-free Dulbecco’s modified eagle medium (DMEM) and Ham’s F-12 Nutrient, supplemented with 15 mM HEPES, 400 ng/mL hydrocortisone, 10% fetal bovine serum (FBS), 2 mM L-glutamine, streptomycin (0.1 mg/mL), and penicillin (100 U/mL). The CAL-27 cell line was maintained in phenol red-free DMEM complemented with 10% FBS, 2 mM L-glutamine, streptomycin (0.1 mg/mL), and gentamycin (100 U/mL). To evaluate the cytotoxic activity of DMU-214, gefitinib (Gef), and the combination of both, the confluent stock cultures of SCC-25 and CAL-27 were detached using a trypsin-EDTA solution and seeded in 96-well plates (Corning Inc., Corning, NY, USA) at a density of 1 × 10^4^ cells/well. After 24 h, the medium was removed, and the cells were treated with the compounds tested from the stock solutions prepared in DMSO (POCH, Gliwice, Poland) at the concentrations presented in Table 2. The final concentration of DMSO in the cell treatment solutions was lower than 0.1%. The control cells were cultured under the same conditions with 0.1% DMSO. After 24, 48, and 72 h of incubation, the solutions were removed, and the mixture of the cell medium and MTT prepared in phosphate-buffered saline (PBS) in a concentration of 5 mg/mL was added. After 2 h of incubation at 37 °C, the solutions were removed, and 200 μL of DMSO was added to each well to dissolve the formazan crystals. The absorbance was measured at 570 nm using an Elx-800 microplate reader (BioTek, Winooski, VT, USA) with a reference wavelength of 650 nm. The concentrations of DMU-214 and gefitinib that were required to assess the half-maximal inhibitory concentration (IC_50_) were evaluated from a plot of percent cell viability versus the logarithm of concentration.

### 4.3. Assessment of Apoptosis and Necrosis Induction

Cell Death Detection ELISA^PLUS^ kit was used to detect apoptosis and necrosis in SCC-25 and CAL-27 cells according to the manufacturer’s protocol. The cells were seeded in a 96-well plate at a density of 1 × 10^4^ cells/well and allowed to attach overnight. On the following day, the medium was removed, and DMU-214 (2.5 µM and 5.0 µM), Gef (15 µM), and their mixture in two concentration ranges: (i) 0.5 µM DMU-214 and 2.5 µM Gef, (ii) 1.0 µM DMU-214 and 5.0 µM Gef were added. Camptothecin (CAM, 5.0 µM) was used as a positive control for apoptosis. After 24 h of incubation, the supernatant and the lysate were placed in streptavidin-coated microtiter plates and incubated with the mixture of anti-histone-biotin, anti-DNA-peroxidase, and incubation buffers. After 2 h, unbound antibodies were disposed during the washing step. Quantification of the nucleosomes was assessed by measuring the optical absorbance at 405 nm and 490 nm using an Elx-800 microplate reader (BioTek, Winooski, VT, USA) against substrate solution as a blank. According to the manufacturer’s protocol, the numbers of apoptotic and necrotic cells were expressed as an enrichment factor (EF). 

### 4.4. Determination of Caspase-8, -9 and Caspase-3/7 Activity

Caspase-8, -9, and -3/7 activity was analyzed using a luminescent Caspase-Glo^®^-8, -9, -3/7 assay kit (Promega Co., Madison, WI, USA) following the manufacturer’s instruction. Briefly, the SCC-25 and CAL-27 cells were placed at a density of 1 × 10^4^ cells/well in 96-well plates. After 24 h, the medium was removed, and DMU-214 (2.5 µM and 5.0 µM), Gef (15 µM), and the mixtures of both compounds: (i) 0.5 µM DMU-214 and 2.5 µM Gef, (ii) 1.0 µM DMU-214 and 5.0 µM Gef were added. On the following day, the medium was removed, and ready-to-use reagents were added. Luminescence was measured using BioTek (Winooski, VT, USA).

### 4.5. Cell Cycle Analysis

For cell cycle analysis, the SCC-25 and CAL-27 cells were incubated with the compounds tested, fixed with 70 % ethanol, and stored at −20 °C until further analysis. Then, the cells were washed with PBS, and the mixture of 10 µg/mL of propidium iodide and RNase A was added. After 30 min of incubation, the fluorescence measurement was conducted (the excitation and emission wavelengths: 535 nm and 617 nm, respectively). The stained cells were evaluated using FACS Canto flow cytometer (Becton-Dickinson, Franklin Lakes, NJ, USA). Data analysis and acquisition were performed using FACS Diva software (Becton-Dickinson, Franklin Lakes, NJ, USA). 

### 4.6. The RNA Isolation and Real-Time Quantitative PCR-Array (qPCR-array) Analysis

The total RNA was isolated using a single-step method by acid guanidinium thiocyanate-phenol-chloroform extraction [32]. The RNA concentration was quantified by measuring the optical density at 260 nm with DeNovix DS-11 spectrophotometer (DeNovix Inc., Wilmington, DE, USA). 1 µg sample of total RNA was reverse-transcribed into cDNA using 4 µL of 2.5 mM dNTP (Roche Diagnostic, Mannheim, Germany), 0.5 µL of oligo(dT) (Novazym, Poznan, Poland) and 0.5 µL of random primers (Roche Diagnostic, Mannheim, Germany). 

RT-qPCR analysis was performed using LightCycler^®^ Instrument 96 (Roche Diagnostic, Mannheim, Germany) and LightCycler Software 1.5. The qPCR-array reaction was carried out on 96-well predesigned EGFR SIGNALING H96 plates (Bio-Rad, Hercules, CA, USA). To each well 1 µL of total cDNA solution, 2 µL of Quantum EvaGreen^®^ PCR Kit, and 7 µL of water were added. The quantity of analyzed cDNA in each sample was standardized by three housekeeping genes, TBP (TATA box binding), GAPDH (glyceraldehyde-3-phosphate dehydrogenase), and HPRT-1 (hypoxanthine phosphoribosyltransferase 1). 

### 4.7. Sodium Dodecyl Sulphate-Polyacrylamide Gel Electrophoresis (SDS-PAGE) and Western Blotting Analysis

The SCC-25 and CAL-27 cells were washed with PBS twice and treated with RIPA lysis buffer and protease inhibitor to perform the protein isolation. Next, 20 μg of protein was resuspended in sample buffer and separated using 4 to 20% gradient Mini-PROTEAN^®^ TGX™ gel (Bio-Rad, Hercules, CA, USA) during SDS-PAGE (parameters: 120 V, 90 min). The protein transfer was performed with the aid of Trans-Blot Turbo Mini 0.2 µm PVDF Transfer Packs set. Immunodetection was performed with rabbit primary EGF Receptor (T 43) Ab (Cell Signaling, #2963), rabbit primary Phospho-EGF Receptor (Tyr1068) (D7A5) XP® Ab (Cell Signaling, #3777) followed by incubation with anti-rabbit secondary IgG, HRP-linked goat Ab (#7074). The PVDF membranes were incubated with anti- β-Actin (13E5) rabbit Ab (HRP Conjugate) (Cell Signaling, #5125). Bands were revealed using Super Signal West Pico PLUS Chemiluminescent Substrate.

## Figures and Tables

**Table 1:** IC_50_ values for DMU-214 and Gef in CAL-27 and SCC-25 cell lines.

Compounds Tested	Incubation Time (h)	IC_50_ Value (μM)	
		CAL-27	SCC-25
DMU-214	24	0.13 ± 0.01	5.31 ± 1.70
	48	0.08 ± 0.01	1.75 ± 0.19
	72	0.07 ± 0.01	1.63 ± 0.17
Gef	24	17.14 ± 5.00	36.15 ± 1.09
	48	2.00 ± 0.03	17.66 ± 3.33
	72	1.03 ± 0.01	8.64 ± 2.67

**Table 2:** Concentrations of DMU-214, Gef, and the mixture of DMU-214 and Gef used in MTT assay.

Cell Line	Compound Tested	Concentration Range [μM]
CAL-27	DMU-214	1; 0.5; 0.25; 0.125; 0.0625; 0.03125
	GEFITINIB	50; 25; 12.5; 6.25; 3.125; 1.5625
	DMU-214 + GEFITINIB	(1 DMU-214 + 50 Gef) (0.5 DMU-214 + 25 Gef) (0.25 DMU-214 + 12.5 Gef) (0.125 DMU-214 + 6.25 Gef) (0.0625 DMU-214 + 3.125 Gef) (0.03125 DMU-214 + 1.5625 Gef)
SCC-25	DMU-214	10; 5; 2.5; 1.25; 0.625; 0.3125
	GEFITINIB	50; 25; 12.5; 6.2 5; 3.125; 1.5625
	DMU-214 + GEFITINIB	(10 DMU-214 + 50 Gef) (5 DMU-214 + 25 Gef) (2.5 DMU-214 + 12.5 Gef) (1.25 DMU-214 + 6.25 Gef) (0.625 DMU-214 + 3.125 Gef) (0.3125 DMU-214 + 1.5625 Gef)

## Data Availability

The data presented in this study are available on request from the corresponding author.
